# *Komagataella phaffii* Cue5 Piggybacks on Lipid Droplets for Its Vacuolar Degradation during Stationary Phase Lipophagy

**DOI:** 10.3390/cells11020215

**Published:** 2022-01-10

**Authors:** Ravinder Kumar, Ankit Shroff, Taras Y. Nazarko

**Affiliations:** 1 Section of Molecular Biology, Division of Biological Sciences, University of California, San Diego, La Jolla, CA 92093, USA; ravind@rpi.edu; 2 Department of Biology, Georgia State University, Atlanta, GA 30303, USA; ashroff@gsu.edu

**Keywords:** CUE, Cue5, *Komagataella phaffii*, lipid droplets, lipophagy, *Pichia pastoris*, Prl1, selective autophagy, stationary phase, yeast

## Abstract

Recently, we developed *Komagataella phaffii* (formerly *Pichia pastoris*) as a model for lipophagy, the selective autophagy of lipid droplets (LDs). We found that lipophagy pathways induced by acute nitrogen (N) starvation and in stationary (S) phase have different molecular mechanisms. Moreover, both types of lipophagy are independent of Atg11, the scaffold protein that interacts with most autophagic receptors and, therefore, is essential for most types of selective autophagy in yeast. Since yeast aggrephagy, the selective autophagy of ubiquitinated protein aggregates, is also independent of Atg11 and utilizes the ubiquitin-binding receptor, Cue5, we studied the relationship of *K. phaffii* Cue5 with differentially induced LDs and lipophagy. While there was no relationship of Cue5 with LDs and lipophagy under N-starvation conditions, Cue5 accumulated on LDs in S-phase and degraded together with LDs via S-phase lipophagy. The accumulation of Cue5 on LDs and its degradation by S-phase lipophagy strongly depended on the ubiquitin-binding CUE domain and Prl1, the positive regulator of lipophagy 1. However, unlike Prl1, which is required for S-phase lipophagy, Cue5 was dispensable for it suggesting that Cue5 is rather a new substrate of this pathway. We propose that a similar mechanism (Prl1-dependent accumulation on LDs) might be employed by Prl1 to recruit another ubiquitin-binding protein that is essential for S-phase lipophagy.

## 1. Introduction

The Cue5 protein belongs to a protein family called CUET. This family includes Cue5 in yeast and toll interacting protein (TOLLIP) in humans. The Cue5 and TOLLIP possess at least two common structural features, the ubiquitin-binding CUE (coupling of ubiquitin conjugation to endoplasmic reticulum (ER) degradation) domain and AIM, the Atg8-interacting motif [1,2]. The CUE domain is structurally similar to the ubiquitin-associated (UBA) domain [3] and enables the proteins to bind a single ubiquitin molecule [4] or polyubiquitin chains [1]. The AIM or LIR (for LC3-interacting region; an acronym commonly used in mammals) is a conserved motif that confers proteins the ability to bind the autophagosomal marker proteins of Atg8 family, e.g., yeast Atg8 or human microtubule associated protein 1 light chain 3 beta (MAP1LC3B) [5]. Proteins that possess both the ubiquitin-binding domain and AIM often act as the ubiquitin-binding autophagic receptors or, simply, “ubiquitin-Atg8 adaptors”, as they facilitate the interaction of ubiquitinated substrates (cargo) with the Atg8-family proteins on the inner surface of autophagosome (vesicular carrier) leading to cargo sequestration and delivery to the vacuole/lysosome for degradation and recycling. Such a type of autophagic degradation where cargo is selected via ubiquitin (or another protein ligand) is termed “selective autophagy” and the selective autophagy of ubiquitinated protein aggregates is called aggrephagy [6]. The CUET proteins serve as ubiquitin-Atg8 adaptors for aggrephagy pathway [1,2]. Besides clearance of protein aggregates, Cue5 adaptor is also involved in the autophagic elimination of inactive 26S proteasomes called proteaphagy [7].

Like aggrephagy and proteaphagy, most of the other selective autophagy pathways have specific proteins that act as their selective autophagy receptors (SARs) [8]. However, yeast SARs other than Cue5 do not bind ubiquitin. Instead, they bind specific protein ligands on cargo surface (e.g., pexophagy SAR, Atg30, binds the peroxisomal membrane proteins, Atg37, Pex3 and Pex14 [9,10,11]) or are anchored in cargo membrane (e.g., mitophagy SAR, Atg32, is inserted in the mitochondrial membrane [12,13]). Additionally, most of yeast SARs (except Cue5) bind the autophagic scaffold protein, Atg11, which is involved in the vast majority of selective autophagy pathways at a step of cargo sequestration [8]. Recently, we developed the *Komagataella phaffii* yeast as a new model for lipophagy, the selective autophagy of lipid droplets (LDs), and showed that, similar to *S. cerevisiae* lipophagy [14,15,16], the lipophagy in *K. phaffii* is independent of Atg11 [17]. Since the same is true for *S. cerevisiae* aggrephagy [1], these pathways must follow a mechanistically different cargo sequestration mechanism. Due to similarities between lipophagy and aggrephagy, we explored the relationship of aggrephagy SAR, Cue5, with LDs and lipophagy.

Here, we used two experimental conditions that induce robust lipophagy in yeast: the acute nitrogen (N) starvation and stationary (S) phase of growth (which is effectively a gradual carbon starvation) [18]. They stimulate the N-starvation and S-phase lipophagy pathways, respectively, with distinct molecular mechanisms [17]. Our previous study identified a unique *prl1* mutant deficient specifically in the S-phase lipophagy [17]. In the present study, we discovered Cue5 as a new S-phase lipophagy substrate. It accumulates on the LDs specifically in the S-phase in the CUE domain- and Prl1-dependent manner. Despite Cue5 itself is not required for the S-phase lipophagy, it uncovers the potential mechanism of action of Prl1 that might operate by promoting recruitment of various ubiquitin-binding proteins to the surface of LDs in S-phase, and at least one of such proteins (which we designated “AtgX”) must be required for the S-phase lipophagy, such as Prl1.

## 2. Materials and Methods

### 2.1. Strains and Plasmids

Table 1 shows the *K. phaffii* wild-type (WT) and mutant strains, as well as plasmids that were used in this study. The pRK2 plasmid with the Erg6-GFP expression cassette was described before [17]. The pRK6 plasmid has the *K. phaffii CUE5* (PAS_chr2-2_0292) deletion cassette, which is released by double digestion with KpnI and SacI. It was built in two steps: first, by inserting the 1000 bp 5′-untranslated region of *CUE5* as KpnI-SalI fragment into the Zeocin^R^ vector, pAP1, to create an intermediate plasmid, pRK5, and then, by inserting the 934 bp 3′-untranslated region of *CUE5* as NotI-SacI fragment into pRK5 to create pRK6. The pRK10 plasmid contains the 347 bp promoter and open reading frame (without STOP codon) of *CUE5* cloned as KpnI-SpeI fragment into an intermediate plasmid, pRK1, that was constructed by inserting the 6xGly-GFP (without first Met) as SphI-SphI fragment into the integrative vector, pIB1 [19]. The pRK24 and pRK25 plasmids are the site-directed mutagenesis products of pRK10. The pRK24 and pRK25 encode the Cue5^CUE^ (F78A, P79A) and Cue5^AIM^ (W308A, Q309A, P310A, L311A) variants of Cue5-GFP, respectively. All the polymerase chain reaction (PCR) products were verified after cloning by sequencing. The *K. phaffii* strains were transformed with plasmids by electroporation [20,21]. The *cue5* deletion mutant was selected as a Zeocin^R^-transformant of PPY12h strain with KpnI- and SacI-digested pRK6 plasmid on YPD + Zeocin plates (10 g/L yeast extract, 20 g/L peptone, 20 g/L dextrose, 20 g/L agar and 100 mg/L Zeocin) and verified by PCR. The plasmids with *HIS4* marker were linearized in the middle of *HIS4* with EcoNI for their integration into *his4* locus. The His^+^-transformants were selected on SD + CSM-His plates and screened for the expression of GFP-fusions by immunoblotting with anti-GFP bodies (11814460001; Roche Diagnostics, Mannheim, Germany), as previously described [17].

### 2.2. Fluorescence Microscopy

The *K. phaffii* cells were imaged as previously described [17]. Briefly, they were grown for 1 d in 1 mL of YPD (1% *w/v* yeast extract, 2% *w/v* peptone and 2% *w/v* dextrose) medium with 1 µL of 1 mg/mL FM 4-64 (T3166; Molecular Probes, Eugene, OR, USA) dissolved in DMSO as the vacuolar membrane stain. During the last hour of incubation, we added 1 µL of 0.1 M monodansylpentane (MDH) (SM1000a; Abcepta, San Diego, CA, USA) to stain the LDs. The 20 µL aliquots were removed and kept on ice before imaging “YPD, 1 d” time-point. The rest of YPD culture was incubated for 2 more days to reach the late S-phase for imaging “YPD, 3 d” time-point. In some experiments, we also removed 3 OD_600_ of cells from “YPD, 1 d” cultures, washed them twice with 1 mL of 1× YNB (1.7 g/L yeast nitrogen base without amino acids and ammonium sulfate), and resuspended in 3 mL of SD-N medium (1× YNB and 20 g/L dextrose). After removing the 1 mL aliquots of “SD-N, 0 h” time-point, the 2 mL SD-N cultures were incubated for 24 h (the last hour with 2 µL of 0.1 M MDH) to generate “SD-N, 24 h” time-point. At each time-point, we immobilized the cells using 1% low-melt agarose and imaged them as previously described [17]. All fluorescence microscopy experiments were performed at least in duplicate.

### 2.3. Biochemical Studies

The *K. phaffii* cells were processed as previously described [17]. Briefly, they were grown in 1 mL YPD medium and equal biomass (1 OD_600_) was taken at time-points 1 d and 3 d for studying S-phase lipophagy. Ponceau S staining was used as a loading control. In some experiments, we also removed 3 OD_600_ of cells from “YPD, 1 d” cultures, washed them twice with 1 mL of 1× YNB, and resuspended in 3 mL of SD-N medium (starting OD_600_ = 1) for studying N-starvation lipophagy. Equal volumes of cultures (1 mL) were taken at time-points 0 h and 24 h in SD-N medium to nullify the differential growth (GFP-fusion dilution) effects of various strains in this medium (loading control not applicable). The samples of cell cultures at all time-points were trichloroacetic acid precipitated [26] and analyzed by immunoblotting with anti-GFP (commercially available; see above) or anti-Ape1 [27] bodies. All biochemical studies were performed at least in duplicate.

### 2.4. Statistical Analysis

For statistical analysis of fluorescence microscopy, we calculated the percentage of cells with at least one instance of Cue5-GFP/MDH co-localization for 10 non-overlapping fields of view (with around 50–100 cells per image) from two independent experiments. To qualify as a dual Cue5-GFP/MDH punctum, the intensity of Cue5-GFP fluorescence at the MDH punctum had to be above the background level of diffuse cytosolic Cue5-GFP fluorescence. Then, we calculated the average and standard error for percentage of cells with Cue5 on LDs for 10 non-overlapping fields of view. Student’s *t*-test (two-tailed distribution, two-sample unequal variance) was used to calculate *p*-values.

## 3. Results

### 3.1. K. phaffii Cue5 Accumulates on LDs Specifically in the Stationary Phase

**Figure 1:**
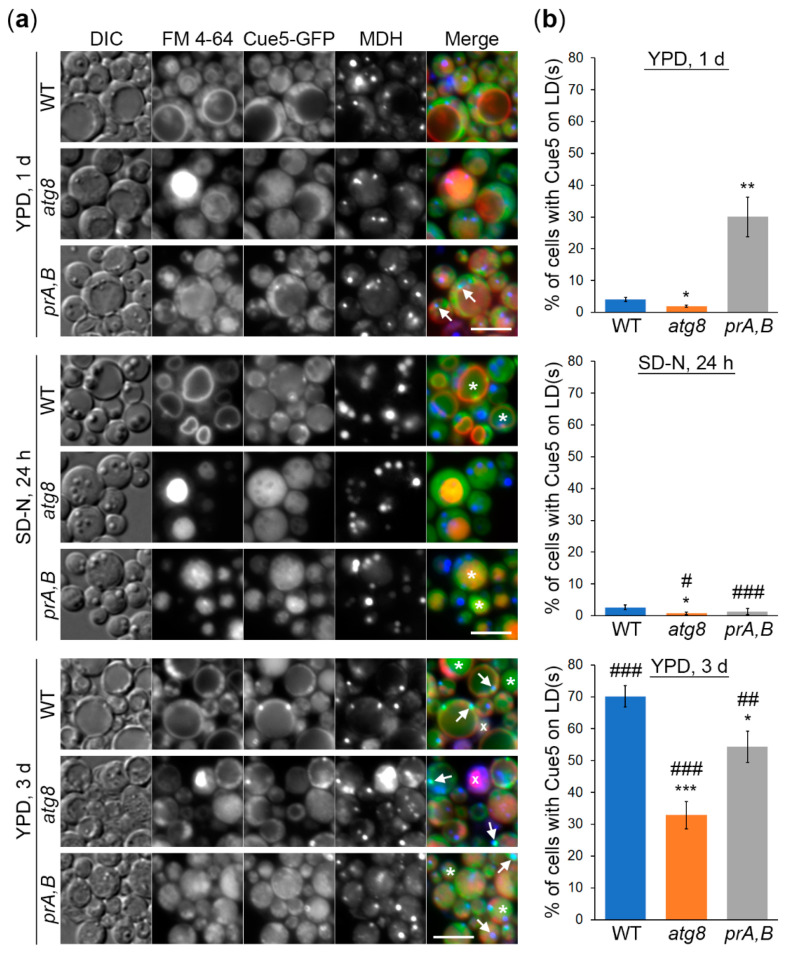
*K. phaffii* Cue5 accumulates on lipid droplets (LDs) specifically in the stationary (S) phase. The cells of wild-type (WT), *atg8* and *prA,B* (*pep4 prb1*) strains that express Cue5-GFP were grown for 1 d in YPD with the vacuolar membrane stain, FM 4-64 (red). During the last hour of growth, LDs were stained blue with monodansylpentane (MDH). One aliquot of cell cultures was imaged immediately after 1 d in YPD (top panels), while another was transferred to SD-N and imaged after 24 h (LDs were stained with MDH during the last hour) of nitrogen (N) starvation (middle panels). The rest of YPD cultures was incubated for 2 more days to reach the late S-phase (bottom panels). (**a**) Representative images. *: Vacuoles with Cue5-GFP; ↑: LDs with Cue5-GFP; x: Dead cell. Scale bar, 5 µm. (**b**) Quantification of images in (**a**). Displayed are the averages and standard errors. * and #: *p* ≤ 0.05; ** and ##: *p* ≤ 0.01; *** and ###: *p* ≤ 0.001; *, ** and ***: mutant versus WT at the same time-point; #, ## and ###: “SD-N, 24 h” or “YPD, 3 d” versus “YPD, 1 d” for the same strain.

Since the SARs tag their substrates for autophagic degradation, we tested if Cue5 would tag LDs under lipophagy conditions. To study the localization of *K. phaffii* Cue5, we expressed the Cue5-GFP fusion protein under *K. phaffii CUE5* promoter in WT, *atg8* and *prA,B* (*pep4 prb1*) strains (Figure 1). Atg8 is a protein that resides on the isolation membrane and completed autophagosome and acts as a binding partner for all the SARs, including Cue5 [1]. Therefore, Cue5 is not associated with any autophagic membranes in *atg8* cells, whereas it is expected to be trapped in the intravacuolar autophagic bodies in protease A and B deficient *pep4 prb1* cells. We found that after 1 d in YPD medium, Cue5-GFP was mostly cytosolic and absent on the MDH-stained LDs (except in *prA,B*, see below), as well as in the FM 4-64-delineated vacuoles (in all strains) (Figure 1a,b, top panels). Transferring the cells to N-starvation conditions led to appearance of Cue5-GFP in the vacuoles of WT and *prA,B* cells (Figure 1a, middle panels) consistent with its role in aggrephagy pathway [1]. The *atg8* cells had fragmented vacuoles in SD-N medium, as expected, which precluded addressing the co-localization of Cue5-GFP with the vacuoles. However, incubating the cells 2 d longer in YPD medium and letting the cultures to enter the late S-phase revealed a similar relocation of Cue5-GFP to the vacuoles in WT and *prA,B*, but not *atg8*, cells, as expected. Remarkably, now, the Cue5-GFP co-localized with LDs in the substantial fraction of cells of all strains (Figure 1a,b, bottom panels). Therefore, we concluded that *K. phaffii* Cue5 accumulates on LDs specifically in the S-phase. The efficiency of this process depends on intact autophagy and vacuolar proteolysis. The reason why *prA,B* cells start accumulating Cue5 on LDs earlier than other strains is probably because they advance to the late S-phase sooner than other cells due to their inability to recycle nutrients in the vacuole.

### 3.2. Cue5 Is Degraded by the Stationary Phase Lipophagy

**Figure 2:**
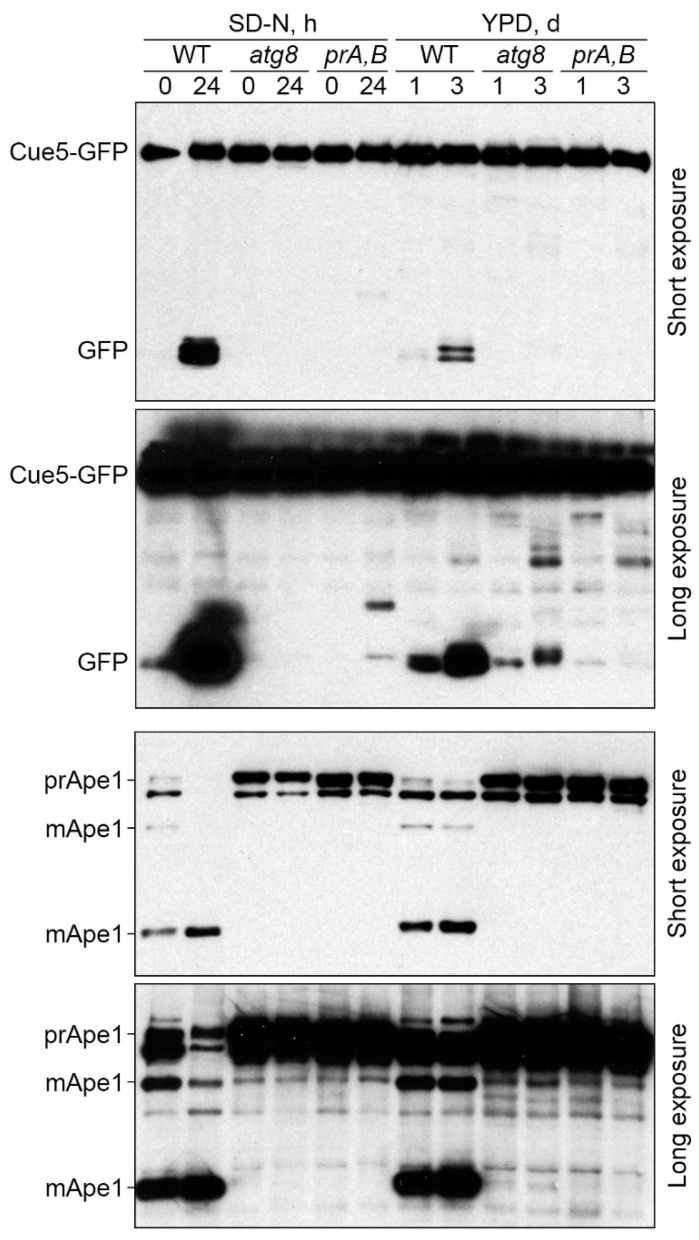
Cue5 is degraded by the S-phase lipophagy. The cells of WT, *atg8* and *prA,B* strains that express Cue5-GFP were grown in YPD medium and equal biomass was taken at time-points 1 d and 3 d for testing S-phase lipophagy. Ponceau S staining (Figure S1) was used as a loading control. A fraction of cells from “YPD, 1 d” cultures was transferred to SD-N medium at OD_600_ = 1 and equal volumes of cultures were taken at time-points 0 h and 24 h for testing N-starvation lipophagy (loading control not applicable). prApe1: precursor form of Ape1; mApe1: mature form of Ape1.

The *K. phaffii* LDs are degraded in S-phase via the process known as S-phase lipophagy [17]. Strikingly, Cue5-GFP localizes to both LDs and vacuoles in S-phase of *K. phaffii* WT and *prA,B* cells (Figure 1a) opening a possibility that Cue5 might be delivered to the vacuole via S-phase lipophagy. To test this hypothesis, we performed the Cue5-GFP processing assay (Figure 2, top panels). In this assay, Cue5-GFP is processed to GFP only after its delivery to the vacuole due to the resistance of GFP to vacuolar proteases. Indeed, acute N-starvation in SD-N medium (control) or reaching the late S-phase in YPD medium induced a robust processing of Cue5-GFP to GFP in WT cells but not in *prA,B* mutant, consistent with the vacuolar degradation of Cue5 under both conditions. If the delivery of Cue5-GFP to the vacuole in S-phase proceeds via S-phase lipophagy, then it should have the same requirements, as the delivery of LD-associated Erg6-GFP that only partially depends on the core autophagic machinery (e.g., Atg8) [17]. Therefore, we tested the Cue5-GFP processing in *atg8* cells. While Cue5-GFP processing fully depended on Atg8 under N-starvation conditions, it only partially relied on Atg8 in S-phase (Figure 2, long exposure for GFP) mimicking the requirement of Erg6-GFP processing [17]. These results suggest that in addition to common localization of Cue5 (Figure 1) and Erg6 [17] to the LDs in S-phase, these proteins also follow the same route to the vacuole.

To test our hypothesis further and exclude a possibility that Atg8 has a partial requirement for all autophagic pathways in S-phase, we blotted the same lysates with the antibodies to Ape1 (Figure 2, bottom panels). Ape1 is a cargo of the cytoplasm-to-vacuole targeting (Cvt) pathway [27]. After delivery to the vacuole, the precursor form of Ape1 (prApe1) undergoes maturation process, which results in the appearance of two mature forms of the protein (mApe1) [24]. As expected, both N-starvation (control) and late S-phase induced maturation of Ape1 in WT, but not *pep4 prb1*, cells in agreement with the vacuolar processing of Ape1 under both conditions. Importantly, the maturation of Ape1 was fully blocked in *atg8* cells under both conditions (Figure 2, long exposure for Ape1). These results suggest that while being partially required for the selective autophagy of LDs [17], Atg8 is essential for the selective autophagy of Ape1 in S-phase. As such, *atg8* mutant can indeed be used to distinguish between the delivery routes to the vacuole in S-phase. Collectively, our results indicate that the LD-associated Cue5, similar to the LD-associated Erg6, is degraded in the vacuole via S-phase lipophagy.

### 3.3. Cue5 Accumulation on LDs and Degradation by S-Phase Lipophagy Depend on CUE Domain

**Figure 3:**
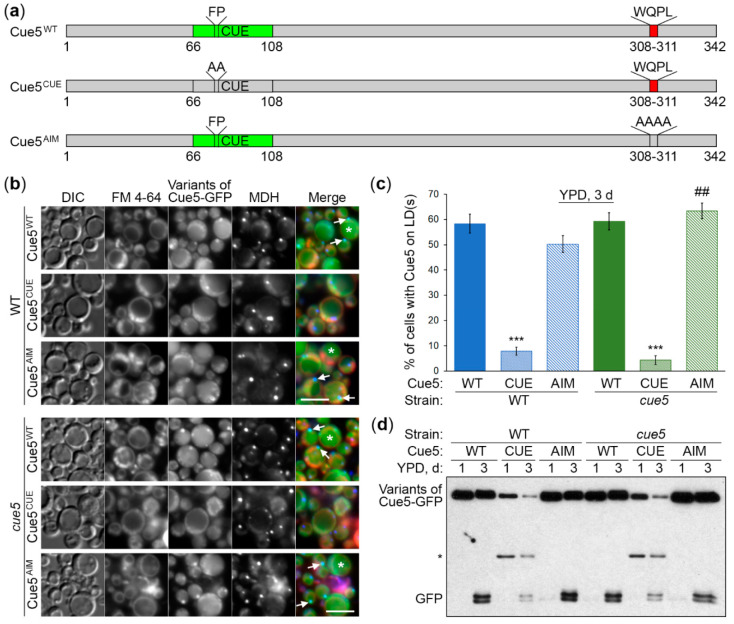
Cue5 accumulation on LDs and degradation by S-phase lipophagy depend on CUE domain. (**a**) The *K. phaffii* Cue5 variants used in this study. CUE: ubiquitin-binding domain; AIM: Atg8-interacting motif. (**b**) Localization of Cue5-GFP variants. The cells of WT and *cue5* strains expressing different variants of Cue5-GFP were grown for 3 d in YPD medium with FM 4-64 (vacuolar membrane, red) and MDH (the last 2 d, LDs, blue). *: Representative vacuoles with Cue5-GFP; ↑: Representative LDs with Cue5-GFP. Scale bar, 5 µm. (**c**) Quantification of images in (**b**). Displayed are the averages and standard errors. ##: *p* ≤ 0.01, *cue5* versus WT strain for the same Cue5-GFP variant; ***: *p* ≤ 0.001, Cue5^CUE^ versus Cue5^WT^ in the same strain. (**d**) Processing of the Cue5-GFP variants to GFP. The cells of WT and *cue5* strains expressing different variants of Cue5-GFP were grown in YPD medium and equal biomass was taken at time-points 1 d and 3 d for testing S-phase lipophagy. Ponceau S staining (Figure S2) was used as a loading control. *: Intermediate GFP-fusion band.

Analysis of the 342 amino acid (aa)-long *K. phaffii* Cue5 sequence with iLIR v1.0 (http://repeat.biol.ucy.ac.cy/iLIR/ (accessed on 21 November 2017)) revealed that similar to *S. cerevisiae* Cue5 [1], it contains the N-terminal ubiquitin-binding domain (CUE, aa 66-108) and C-terminal Atg8-interacting motif (AIM, aa 308-311, WxxL). Since the *S. cerevisiae* Cue5^CUE^ (F109A, P110A) and Cue5^AIM^ (W373A, Q374A, P375A, L376A) variants were unable to bind the ubiquitin conjugates and Atg8, respectively [1], we created the corresponding variants of *K. phaffii* Cue5, Cue5^CUE^ (F78A, P79A) and Cue5^AIM^ (W308A, Q309A, P310A, L311A) (Figure 3a), and addressed their effects on the recruitment of Cue5-GFP to LDs in S-phase and its degradation with LDs via S-phase lipophagy (Figure 3b–d).

The *K. phaffii* Cue5-GFP fusion with the WT Cue5 (Cue5^WT^) variant displayed a co-localization with LDs in more than half of WT cells after their incubation for 3 d in YPD medium, as observed earlier. While the Cue5^AIM^ variant displayed the same pattern of localization as Cue5^WT^, the Cue5^CUE^ variant was severely affected in its ability to accumulate on LDs in S-phase (Figure 3b, top panels). Quantification of images confirmed these observations (Figure 3c, blue columns) suggesting that CUE domain plays an essential role in the recruitment of Cue5 to LDs. We also noticed that Cue5^WT^ and Cue5^AIM^ variants of Cue5-GFP had a higher incidence of localization inside the vacuole lumen compared to Cue5^CUE^ variant. Therefore, we followed up with the Cue5-GFP processing assay that measures vacuolar delivery and degradation (Figure 3d). Indeed, the Cue5^CUE^ variant of Cue5-GFP generated much less free GFP than Cue5^WT^ and Cue5^AIM^ variants in WT background (Figure 3d, left side). Instead, the Cue5^CUE^-GFP might have been cleaved by some cytosolic protease that generated an intermediate GFP-fusion band (marked by an asterisk). In conclusion, CUE domain is required for both accumulation of Cue5 on LDs and its subsequent degradation via S-phase lipophagy.

Recently, it was reported that *S. cerevisiae* Cue5 can self-interact via its CUE domain [28]. Since oligomerization of the *K. phaffii* Cue5^AIM^-GFP with endogenous Cue5 in WT strain could potentially explain normal processing of Cue5^AIM^-GFP via S-phase lipophagy (Figure 3d, left side), we created the *cue5* deletion mutant and used it as an alternative background strain (Figure 3b–d). The Cue5^AIM^-GFP localized to the S-phase LDs in *cue5* cells (even slightly better than in WT background), while the Cue5^CUE^-GFP was unable to do so (Figure 3b, bottom panels; Figure 3c, green columns) confirming the essential role of CUE domain in the association of Cue5 with LDs. Surprisingly, the Cue5^AIM^-GFP was still efficiently processed to GFP in the absence of endogenous Cue5, unlike Cue5^CUE^-GFP (Figure 3d, right side), suggesting that Cue5 AIM motif is dispensable for the S-phase lipophagy. Together, we found that CUE domain is important for Cue5 accumulation on LDs and, consequently, its degradation by the S-phase lipophagy. In contrast, AIM motif is unnecessary not only for Cue5 enrichment on LDs (as expected), but also for S-phase lipophagy, raising the question as to whether Cue5 plays any role in this process (see Section 3.5 below).

### 3.4. Cue5 Accumulation on LDs and Degradation by S-Phase Lipophagy Depend on Prl1

**Figure 4:**
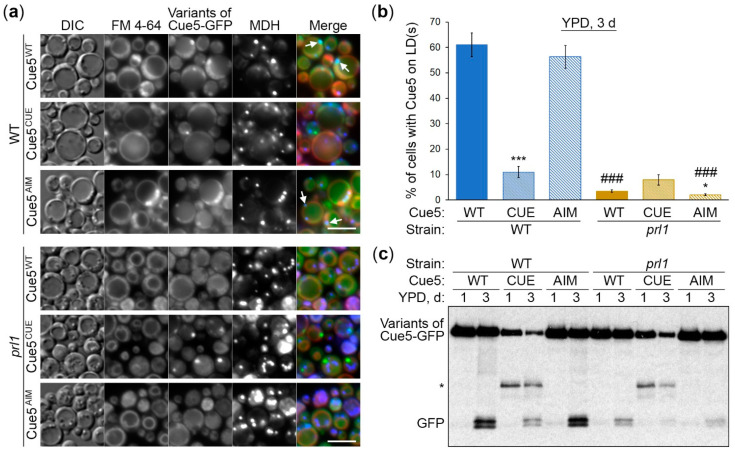
Cue5 accumulation on LDs and degradation by S-phase lipophagy depend on Prl1. (**a**) Localization of Cue5-GFP variants. The cells of WT and *prl1* strains expressing different variants of Cue5-GFP were grown for 3 d in YPD medium with FM 4-64 (vacuolar membrane, red) and MDH (the last 2 d, LDs, blue). ↑: Representative LDs with Cue5-GFP. Scale bar, 5 µm. (**b**) Quantification of images in (**a**). Displayed are the averages and standard errors. * and ***: *p* ≤ 0.05 and *p* ≤ 0.001, for mutant Cue5-GFP versus WT Cue5-GFP in the same strain; ###: *p* ≤ 0.001, *prl1* versus WT for the same Cue5-GFP variant. (**c**) Processing of the Cue5-GFP variants to GFP. The cells of WT and *prl1* strains expressing different variants of Cue5-GFP were grown in YPD medium and equal biomass was taken at time-points 1 d and 3 d for testing S-phase lipophagy. Ponceau S staining (Figure S3) was used as a loading control. *: Intermediate GFP-fusion band.

Previously, we isolated the *K. phaffii prl1* mutant that was specifically affected in lipophagy in the S-phase [17]. Since the accumulation of *K. phaffii* Cue5 on LDs is also specific for S-phase (Figure 1), we studied the relationship between the two proteins. For this, we transformed the Cue5-GFP variants into *prl1* strain and tested their recruitment to LDs and degradation with LDs by lipophagy in the S-phase (Figure 4). The control part of the experiment with the Cue5-GFP variants in WT strain worked as earlier, reiterating a strong requirement of CUE domain for both localization of Cue5 to LDs and its degradation in the vacuole via S-phase lipophagy (Figure 4, WT strain). Interestingly, neither of the Cue5-GFP variants accumulated on LDs in the *prl1* mutant (Figure 4a, bottom panels). This was confirmed by quantification of images (Figure 4b, golden columns). As a result, the processing of Cue5-GFP variants to GFP was also severely affected in *prl1* cells with *prl1* and *cue5^CUE^* mutations having an additive effect (Figure 4c, right side). These results suggest that similar to CUE domain, Prl1 is required for the recruitment of Cue5 to LDs and its degradation via S-phase lipophagy.

### 3.5. Cue5 Is Dispensable for S-Phase Lipophagy

**Figure 5:**
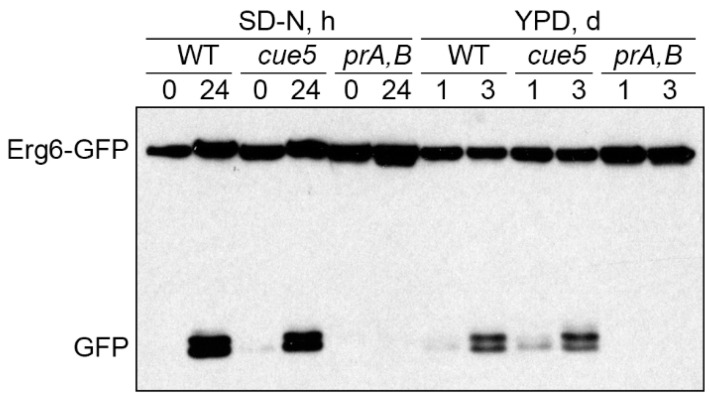
Cue5 is dispensable for S-phase lipophagy. The WT, *cue5* and *prA,B* cells expressing Erg6-GFP were grown in YPD medium and equal biomass was taken at 1 d and 3 d for testing S-phase lipophagy. Ponceau S staining (Figure S4) was used as a loading control. A fraction of cells from “YPD, 1 d” cultures was transferred to SD-N medium at OD_600_ = 1 and equal volumes of cultures were taken at 0 h and 24 h for testing N-starvation lipophagy (loading control not applicable).

Since our results indicated that AIM motif of *K. phaffii* Cue5 is not required for S-phase lipophagy (Figure 3d, right side), we wanted to address the question if *K. phaffii* Cue5 plays a role in this process. Recently, it was reported that *S. cerevisiae* Cue5 is not required for S-phase lipophagy [29] making such a possibility in *K. phaffii* even more likely. To test lipophagy in *K. phaffii cue5* mutant, we used a previously described Erg6-GFP processing assay [17]. As predicted by the non-LD localization of Cue5 under N-starvation conditions (Figure 1a, middle panels), the *cue5* mutant was proficient in the Erg6-GFP processing to GFP relative to WT and *prA,B* strains in SD-N medium (Figure 5, left side). However, the same was also true for *cue5* and Erg6-GFP processing in S-phase (Figure 5, right side) suggesting that *K. phaffii* Cue5 is dispensable for the S-phase lipophagy despite accumulation of the protein on the LDs in S-phase.

## 4. Discussion

**Figure 6:**
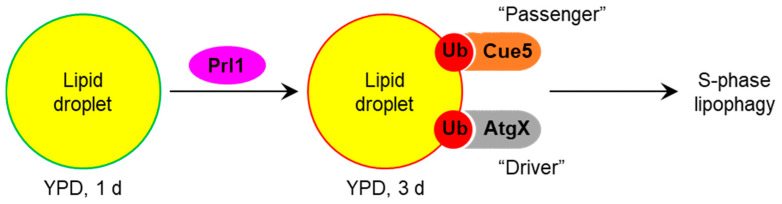
Relationship of *K. phaffii* Cue5 with LDs and lipophagy. In S-phase, Cue5 accumulates on LDs and is degraded together with LDs via S-phase lipophagy. The accumulation of Cue5 on LDs and its vacuolar degradation strongly depend on the ubiquitin-binding CUE domain and Prl1, the positive regulator of lipophagy 1. Since Cue5 is dispensable for the S-phase lipophagy, it is rather a new “passenger” of this trafficking route. However, a similar mechanism might be employed by Prl1 to recruit another ubiquitin-binding protein (AtgX) that will “drive” S-phase lipophagy.

In this study, we explored the relationship between the *K. phaffii* Cue5 protein and LDs, especially their autophagic turnover by lipophagy (reviewed in [18]). Since *S. cerevisiae* Cue5 is the established SAR for aggrephagy and proteaphagy [1,7], and both lipophagy and aggrephagy are independent of the key selectivity factor, Atg11, in yeast [14,15,16,17], we entertained the possibility of Cue5 playing a role of the SAR for lipophagy. A careful analysis of Cue5 localization showed that Cue5 can indeed tag LDs under certain conditions, such as the S-phase of growth (Figure 1). Moreover, Cue5 follows LDs to the vacuole for autophagic degradation during the S-phase lipophagy (Figure 2). The structure–function analysis of Cue5 indicated that the ubiquitin-binding CUE domain (but not the Atg8-interacting AIM motif) is essential for the correct localization of Cue5 to LDs and its subsequent degradation by lipophagy in S-phase (Figure 3). Interestingly, the accumulation of Cue5 on LDs and its vacuolar degradation also strongly depend on the S-phase lipophagy-specific factor, Prl1 [17] (Figure 4). Despite Prl1 promoting the localization of Cue5 to LDs in S-phase, Cue5 is not its lipophagy effector, because Cue5 is dispensable for the S-phase lipophagy (Figure 5). Instead, Cue5 is a new “passenger” of the S-phase LDs with an as yet unknown “driver” that takes them to the vacuole for degradation and recycling (Figure 6).

We also found that the efficiency of Cue5 recruitment to LDs in S-phase depends on Atg8, the protein marker of autophagic membranes that binds all the SARs, including Cue5 [1] (Figure 1b, bottom panel). However, we can exclude a direct role of Atg8 in the localization of Cue5 to the S-phase LDs, because the Cue5^AIM^-GFP variant with mutated AIM motif was characterized by normal or even slightly elevated accumulation on LDs compared to Cue5^WT^-GFP variant in the WT or *cue5* background, respectively (Figure 3c). Since (in addition to *atg8*) a similar partial defect of Cue5 recruitment to LDs was also observed in *prA,B* cells (Figure 1b, bottom panel), we favor a scenario where intact autophagy and vacuolar proteolysis have an indirect positive effect on this process (e.g., by supporting the energy demanding ubiquitination of LD proteins in S-phase via degradation of intracellular macromolecules).

We present two independent lines of evidence that Cue5 is degraded together with LDs in S-phase. First, the requirement of the vacuolar degradation of Cue5 mimics the specific requirement of the vacuolar turnover of LD-associated Erg6 [17]. Both protein degradation pathways only partially rely on the core autophagic machinery represented by Atg8, while Atg8 is essential for the vacuolar processing of non-LD substrates, such as prApe1 (Figure 2). This suggests that Cue5 is co-degrading with the LDs in S-phase. The second line of evidence comes from our mutational analysis. The point mutations in CUE domain that disrupt the accumulation of Cue5^CUE^-GFP variant on LDs in S-phase also disrupt its co-degradation with LDs during lipophagy (Figure 3). Therefore, Cue5 piggybacks on LDs for its vacuolar degradation during S-phase lipophagy. The reason why Cue5 follows this route is not clear but a similar phenomenon was described for several ER proteins during the ER stress-induced lipophagy [30,31]. Therefore, we hypothesize that Cue5 might aggregate the ubiquitinated ER proteins at the surface of LDs. To our knowledge, this is the first report of the ubiquitin-binding SAR using LDs as its carriers for vacuolar delivery, and not contributing anything to the delivery process. Since the ubiquitin-Atg8 adaptors are more common in mammals than in yeast (and not limited to the Cue5 homolog, TOLLIP), care must be taken in evaluating their roles in lipophagy, because some of them might also “stick” to the LDs as “passengers”.

To qualify as a SAR of a given selective autophagy pathway, the protein must not only tag the cargo to be degraded and degrade together with it in the vacuole/lysosome, it must also bridge the cargo with the autophagic membrane via the SAR-Atg8 interaction and both the SAR and its interaction with Atg8 must be essential for degradation [8]. The *S. cerevisiae* Cue5 meets all the above as a SAR for aggrephagy [1]. However, the *K. phaffii* Cue5 satisfies only the first two criteria (cargo tagging and vacuolar turnover with the cargo) of a SAR for lipophagy (Figure 1 and Figure 2), the criteria that are common for the SARs and other cargo-associated proteins. Importantly, the Cue5^AIM^-GFP variant has a normal vacuolar degradation relative to Cue5^WT^-GFP in both the WT and *cue5* backgrounds (Figure 3d) suggesting that the Cue5′s AIM motif is dispensable for the S-phase lipophagy. Moreover, the entire Cue5 is dispensable for the S-phase lipophagy, because *cue5* mutant has normal degradation of the LD-associated Erg6 (Figure 5). Therefore, we conclude that *K. phaffii* Cue5 is a new LD-associated protein and lipophagy substrate in S-phase and not the S-phase lipophagy SAR (denoted as AtgX in Figure 6).

The key to finding the S-phase lipophagy SAR might lie in *prl1*, the S-phase lipophagy-specific mutant [17] that is blocked in the recruitment of Cue5-GFP variants to LDs (Figure 4). We speculate that Prl1 promotes the ubiquitination of LDs in S-phase attracting the ubiquitin-binding proteins, such as Cue5, to their surface (Figure 6). One such protein (AtgX) might prove to be essential for lipophagy, as Prl1.

## Figures and Tables

**Table 1:** *K. phaffii* strains and plasmids used in this study.

Mutant	Strain	Background	Genotype and Plasmid	Source
WT	GS115	GS115	*his4*	[22]
WT	SRK69	GS115	*his4::pRK10 (P_CUE5_-CUE5-GFP, HIS4)*	This study
WT	SRK160	GS115	*his4::pRK24 (P_CUE5_-cue5^CUE^-GFP, HIS4)*	This study
WT	SRK162	GS115	*his4::pRK25 (P_CUE5_-cue5^AIM^-GFP, HIS4)*	This study
WT	PPY12h	PPY12h	*arg4 his4*	[23]
WT	SRK8	PPY12h	*his4::pRK2 (P_ERG6_-ERG6-GFP, HIS4)*	[17]
*atg8*	SJCF925	PPY12h	∆*atg8*::*Geneticin^R^ arg4 his4*	[24]
*atg8*	SRK87	SJCF925	*his4::pRK10 (P_CUE5_-CUE5-GFP, HIS4)*	This study
*cue5*	SRK196	PPY12h	∆*cue5::Zeocin^R^ (pRK6) arg4 his4*	This study
*cue5*	SRK201	SRK196	*his4::pRK2 (P_ERG6_-ERG6-GFP, HIS4)*	This study
*cue5*	SRK223	SRK196	*his4::pRK10 (P_CUE5_-CUE5-GFP, HIS4)*	This study
*cue5*	SRK225	SRK196	*his4::pRK24 (P_CUE5_-cue5^CUE^-GFP, HIS4)*	This study
*cue5*	SRK227	SRK196	*his4::pRK25 (P_CUE5_-cue5^AIM^-GFP, HIS4)*	This study
*pep4 prb1*	SMD1163	GS115	*pep4 prb1 his4*	[25]
*pep4 prb1*	SRK35	SMD1163	*his4::pRK2 (P_ERG6_-ERG6-GFP, HIS4)*	[17]
*pep4 prb1*	SRK71	SMD1163	*his4::pRK10 (P_CUE5_-CUE5-GFP, HIS4)*	This study
*prl1*	SRK3	PPY12h	*prl1::Zeocin^R^ (pRK6) arg4 his4*	[17]
*prl1*	SRK166	SRK3	*his4::pRK10 (P_CUE5_-CUE5-GFP, HIS4)*	This study
*prl1*	SRK168	SRK3	*his4::pRK24 (P_CUE5_-cue5^CUE^-GFP, HIS4)*	This study
*prl1*	SRK170	SRK3	*his4::pRK25 (P_CUE5_-cue5^AIM^-GFP, HIS4)*	This study

## Supplementary Materials

The following are available online at https://www.mdpi.com/article/10.3390/cells11020215/s1, Figure S1: Supplementary figure for Figure 2, Figure S2: Supplementary figure for Figure 3, Figure S3: Supplementary figure for Figure 4, Figure S4: Supplementary figure for Figure 5.
