# Supplementary material for: Komagataella phaffii Cue5 Piggybacks on Lipid Droplets for Its Vacuolar Degradation during Stationary Phase Lipophagy
Source: Cells. 2022 Jan 10;11(2):215. doi: 10.3390/cells11020215 (PMC8774080; doi:10.3390/cells11020215)
Supplement: Supplementary file 1 [file cells-11-00215-s001.zip › cells-1534503-supplementary.pdf]

Supplementary Materials

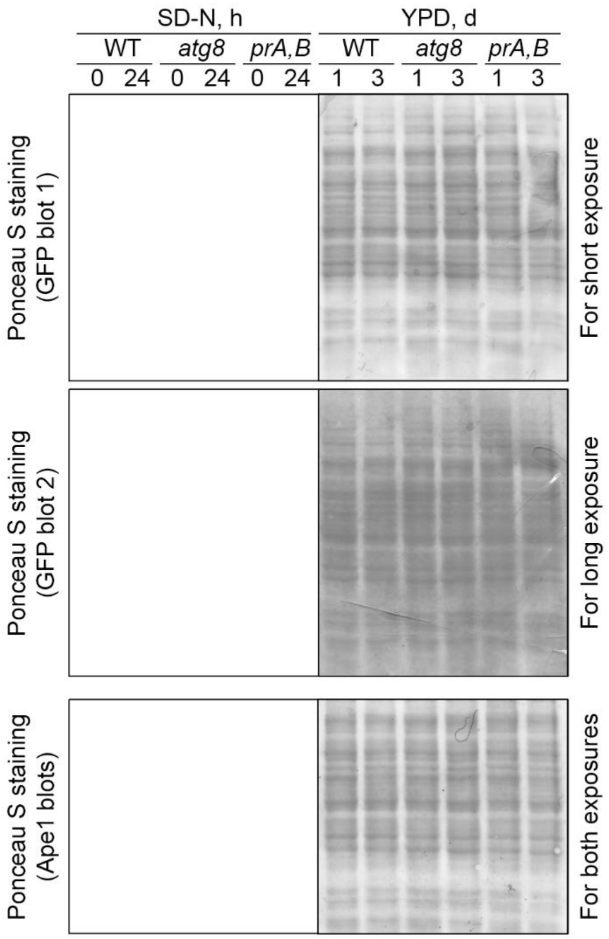

Figure S1. Supplementary figure for Figure 2. Ponceau S staining (loading control for the S-phase lipophagy portions of the membranes). The SD-N portions were intentionally left blank as loading control was not applicable there (see Figure 2 legend for details).

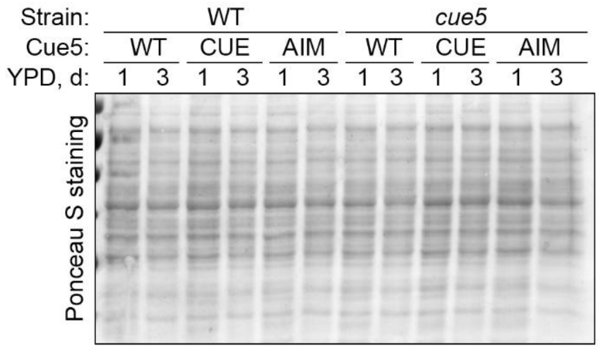

Figure S2. Supplementary figure for Figure 3. Ponceau S staining (loading control). See the legend of Figure 3 for details.

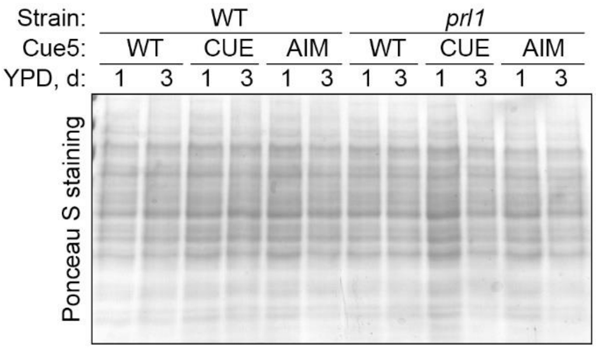

Figure S3. Supplementary figure for Figure 4. Ponceau S staining (loading control). See the legend of Figure 4 for details.

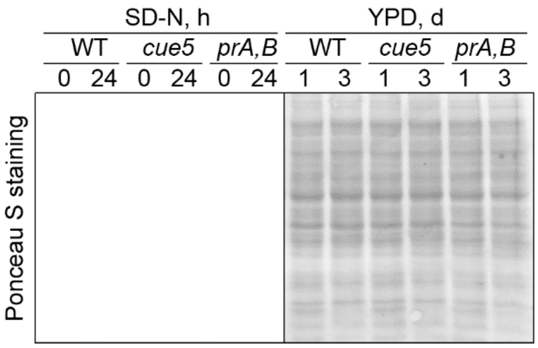

Figure S4. Supplementary figure for Figure 5. Ponceau S staining (loading control for the S-phase lipophagy portion of the membrane). The SD-N portion was intentionally left blank as loading control was not applicable there (see Figure 5 legend for details).
